# Breast Cancer Surgery Wait Times and COVID-19: A Canadian Population-Based Analysis

**DOI:** 10.1245/s10434-025-19029-3

**Published:** 2026-01-25

**Authors:** Gary Ko, Qing Li, Ning Liu, Eitan Amir, Andrea M. Covelli, Antoine Eskander, Vivianne Freitas, C. Anne Koch, Jenine Ramruthan, Emma Reel, Amanda Roberts, Toni Zhong, Tulin D. Cil

**Affiliations:** 1 Division of General Surgery, Department of Surgery, University of Toronto, Toronto, ON Canada; 2 ICES, Toronto, ON Canada; 3 Division of Medical Oncology and Hematology, Department of Medicine, Princess Margaret Cancer Centre, University Health Network, Toronto, ON Canada; 4 Division of Surgical Oncology, Department of Surgery, Mount Sinai Hospital, Toronto, ON Canada; 5 Institute for Health Policy, Management, and Evaluation, University of Toronto, Toronto, ON Canada; 6 Division of Surgical Oncology, Sunnybrook Health Sciences Centre, Toronto, ON Canada; 7 Department of Otolaryngology-Head and Neck Surgery, Sunnybrook Health Sciences Centre, Toronto, ON Canada; 8 Joint Department of Medical Imaging, University of Toronto, Toronto, ON Canada; 9 Radiation Medicine Program, Princess Margaret Cancer Centre, University Health Network, Toronto, ON Canada; 10 Division of General Surgery, Department of Surgery, Princess Margaret Cancer Centre, University Health Network, Toronto, ON Canada; 11 Division of Plastic Surgery, Department of Surgery, University of Toronto, Toronto, Canada

**Keywords:** Breast cancer, Surgery, COVID-19, Pandemic, Wait times, Ontario, NAC, NET

## Abstract

**Background:**

During the COVID-19 pandemic, operating room restrictions led to triaging of breast cancer (BC) surgery. This study assessed the impact of the pandemic on wait times for BC surgery at a population level.

**Methods:**

Patients diagnosed with BC between January 2018 and December 2021 in Ontario, Canada, were categorized as pre-pandemic (January 1, 2018, to March 14, 2020), immediate (March 15, 2020, to June 13, 2020), and peri-pandemic (June 14, 2020, to December 31, 2021). Time-to-event analysis evaluated the time to first BC surgery, with subgroup analyses for patients receiving neoadjuvant chemotherapy (NAC) and neoadjuvant endocrine therapy (NET).

**Results:**

Among 37,520 patients with newly diagnosed BC, the probability of undergoing surgery each month from their diagnosis date was significantly lower during the immediate and peri-pandemic period than during the pre-pandemic period (log-rank *p* < 0.01). However, the use of NAC and NET increased during the immediate pandemic compared with the pre-pandemic (NAC: 24.3% vs. 14.4%; NET 12.4% vs. 6.6%; *p* < 0.01). Among patients who had surgery as first treatment, the median wait time was shortest during the immediate pandemic (1.0 mo) compared with the pre- and peri-pandemic periods (1.2 mo, *p* < 0.01).

**Conclusion:**

This population-based study found longer diagnosis-to-surgery times during the immediate pandemic, with increased use of neoadjuvant therapy. However, surgical wait times (e.g. post NAC) were shorter in the immediate pandemic, highlighting the triaging of BC care during this unprecedented event.

**Supplementary Information:**

The online version contains supplementary material available at 10.1245/s10434-025-19029-3.

## Background

During the initial months of the COVID-19 pandemic, breast cancer (BC) screening was suspended^1^ and there were restrictions in operating room capacity as hospitals diverted resources to treat acutely ill patients. This resulted in delays in the diagnosis and treatment of BC. In response to the pandemic, international cancer organizations, including the American Society for Breast Surgeons and the Society of Surgical Oncology, developed guidelines for triaging patients.^2–4^ Patients who received neoadjuvant chemotherapy (NAC) were prioritized to undergo surgery, and patients with early-stage hormone receptor-positive tumors who would have otherwise had upfront surgery in the pre-pandemic era, notably those with early-stage hormone-positive BC, were placed on neoadjuvant endocrine therapy (NET) and had delayed surgical therapy.^2–4^

Previous studies have demonstrated that delays in BC treatment, including time to surgery, may have detrimental effects on oncologic outcomes, with some showing delays of > 60 days to be associated with worse BC-specific survival.^5,6^ A meta-analysis of 12 studies with over 1,047,384 patients showed that delaying surgery by 12 weeks from the time of BC diagnosis resulted in decreased overall survival, and the impact on survival was most pronounced for patients with stage 1 and 2 disease.^7^ Timing for surgery is even more critical for patients with BC undergoing NAC. A meta-analysis including 8794 patients showed improved overall and disease-free survival among patients who had surgery within 4–8 weeks from the date they completed NAC compared with patients who had surgery < 4 weeks or > 8 weeks from their chemotherapy completion date.^8^ Surgical delays beyond 8 weeks after completion of chemotherapy have been associated with worse survival.^6^

These data demonstrate that delays in time to surgery, from either diagnosis or upon completion of NAC, significantly affect survival outcomes. The purpose of this study was to evaluate the impact of pandemic-related health services changes on operative wait times for patients with BC in Ontario.

## Methods

### Study Design

We conducted a retrospective population-based cohort study using administrative databases stored at the Institute for Clinical Evaluative Sciences (ICES) in Ontario, Canada. Under the Canada Health Act, Ontario’s 14 million residents receive universally accessible and publicly funded health care through the Ontario Health Insurance Plan (OHIP). ICES is an independent, non-profit research institute whose legal status under Ontario’s health information privacy law allows it to collect and analyze health care and demographic data, without consent, for health system evaluation and improvement. This study was reported following the Reporting of Studies Conducted using Observational Routinely Collected Health Data statement.^9^ The use of data in this project is authorized under section 45 of Ontario’s Personal Health Information Protection Act and does not require review by a research ethics board.

### Data Sources

The Ontario Cancer Registry is a provincial database including all patients with a cancer diagnosis (excluding non-melanoma skin cancer) in Ontario since 1964 and was used to identify incident BC cases, including biomarker status and stage data. The Registered Persons Database is a population-based registry maintained by the Ontario Ministry of Health and Long-Term Care that collects information on all individuals covered under OHIP and was used to obtain demographic data and vital status.^10^ The following databases were used to collect information on health services received: Canadian Institute of Health Information Discharge Abstract Database for acute inpatient hospitalizations; the National Ambulatory Care Reporting System for same-day surgery admissions, emergency room visits, and oncology clinic visits; and the OHIP Claims Database for billing from health care providers, including physicians, groups, laboratories, and out-of-province providers. The Cancer Activity Level Reporting database was used to determine which chemotherapeutics and other medications were administered to patients. Dataset details are available in Supplementary Table 1.

Datasets were linked using unique encoded identifiers and analyzed at ICES. The analyst (QL) had complete access to all anonymized datasets used in this study to create the study cohorts, perform data linkage, and conduct analyses.

### Study Population and Cohort

We identified patients with a new diagnosis of BC in the Ontario Cancer Registry using International Classification of Diseases for Oncology (ICD-O.3) codes between January 1, 2018, and December 31, 2021. Patients were included if they had an ICD-behaviour 3 (breast cancer) code. We excluded patients with stage 0 BC (ductal carcinoma in situ) as this was not reliably captured in these datasets. Disease stage data were obtained through Cancer Care Ontario through the collaborative staging data collection system, which collects data on tumor size, number of positive lymph nodes, and involvement of specific tissues or adjacent structures and generates a “best stage” through a computer algorithm combining clinical and pathologic data.^11^ As stage data were not available for the latter part of our study period, we did not set limits with respect to stage (i.e. patients with stage 4 BC were also included). Patients whose date of death preceded the date of diagnosis were excluded. Patients who were diagnosed on the date of their surgery were excluded.

### Exposure

The date of BC diagnosis was the primary exposure of interest. Three time periods were defined: pre-pandemic/baseline (January 1, 2018, to March 14, 2020), immediate pandemic (March 15, 2020, to June 13, 2020), and peri-pandemic (June 14, 2020, to December 31, 2021). March 15, 2020, was designated as the start of the immediate COVID-19 pandemic period as it corresponded to when hospitals and health care facilities were directed to reduce elective surgeries and other non-urgent activities.^11^ June 13, 2020, was identified as the end of the immediate pandemic period, which coincided with the resumption of health care services under precautionary measures.^12^

### Outcome Measures

The primary outcome was the time to first BC surgery, which was determined through OHIP billing codes. Patients were followed from date of diagnosis to date of first surgery, death, 12 months after diagnosis, or March 31, 2023, whichever came first. Only the date of first surgery was counted, as patients could have had multiple BC surgeries for curative intent.

### Covariates

Age and sex were obtained from the Registered Persons Database.^11^ Biomarker status was subdivided into three categories based on estrogen receptor (ER), progesterone receptor (PR), and human epidermal growth factor receptor 2 (HER2) status: (1) hormone receptor (either/both of ER and PR) positive and HER2 negative, (2) HER2 positive, (3) triple negative, or (4) missing. An “unknown” category was created for each variable. We categorized patients into three groups based on their first treatment after their diagnosis date. Those defined as undergoing NAC received chemotherapy regimens as their first therapy after their BC diagnosis. Those classified as NET were patients whose first therapy after diagnosis was endocrine therapy based on Activity Level Reporting data. Patients having upfront surgery were those with a first record of surgical therapy with no record of NAC or NET.

### Statistical Analysis

Descriptive statistics were used to define baseline characteristics of patients diagnosed with BC in each time period. Categorical variables are reported as absolute numbers (n) and proportions (%); continuous variables are reported as medians with interquartile ranges (IQRs). Chi-squared tests were used to compare categorical variables, and analysis of variance (mean)/Kruskal–Wallis test for medians were used to compare continuous variables, as appropriate. Time-to-event analysis was conducted for time to a patient’s first BC surgery. We then conducted subgroup analyses and evaluated treatment times for patients in the three categories of NAC, NET, and upfront surgery. We determined the probability of surgery within 8 weeks from date of diagnosis to encompass time from diagnosis, referral, and consultation using Kaplan–Meier estimates. For patients undergoing neoadjuvant systemic therapy (endocrine therapy and chemotherapy), we selected a time period of 12 months to encompass diagnosis, referral to medical oncology, time for completion of chemotherapy, and unexpected delays during treatment. We did a subgroup analysis and calculated the median wait times for patients who underwent surgery subdivided by their first treatment.

All analyses were performed using SAS Enterprise Guide, version 7.1 (SAS Institute, Cary, NC, USA). Results were considered statistically significant if *p* < 0.05. No adjustment was applied for multiple significance testing. We calculated the median time from date of diagnosis to date of surgery.

## Results

**Fig. 1:**
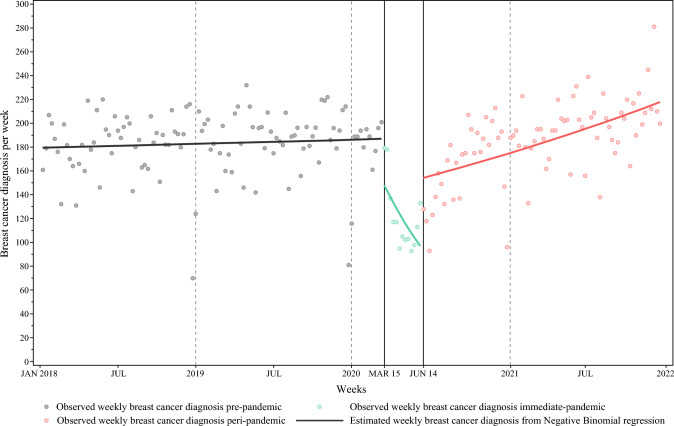
Weekly breast cancer diagnoses in Ontario Canada between January 1, 2018, and December 31, 2021

The study cohort consisted of 37,520 patients with a new diagnosis of BC between January 1, 2018, and December 31, 2021. Figure 1 shows the weekly BC diagnoses in Ontario, Canada between January 1, 2018, and December 31, 2021. The weekly number of BC diagnoses in Ontario decreased by 18.0% during the immediate pandemic compared with pre-pandemic levels (153.2 vs. 179.2; *p* = 0.04). The BC diagnoses recovered to pre-pandemic levels by June 2021.

**Fig. 2:**
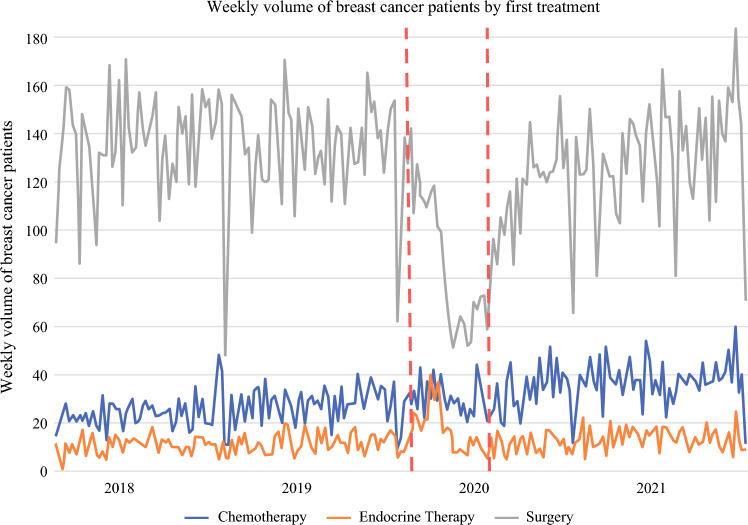
Weekly volume of patients with breast cancer by first treatment over time

The characteristics of patients diagnosed with BC in each period are presented in Table 1. During the immediate pandemic period, there was a lower proportion of patients with stage I BC (pre-pandemic 65.5%; immediate 58.2%; and peri-pandemic 61.5%; *p* < 0.01) and a higher proportion of patients with triple negative BC (pre-pandemic 9.4%; immediate 11.3%; peri-pandemic 9.4%; *p* < 0.01) and HER2+ BC (pre-pandemic 14.2%; immediate 16.6%; peri-pandemic 14.5%; *p* < 0.01). The percentage of patients who had NAC or NET as their first treatment during the immediate pandemic period (NAC 381 [24.3%], NET 195 [12.4%]) was higher than during the pre-pandemic period (NAC 3034 [14.4%], NET 1388 [6.6%]). In the peri-pandemic period, the percentage of patients on NET returned to baseline. Figure 2 shows the weekly volume of patients with BC by first treatment over time. During the immediate pandemic period, the number of patients who had surgery as the first treatment decreased, and the number of patients who had NAC and NET increased, compared with the pre-pandemic period. In the peri-pandemic period, the weekly volume of patients who received NAC was higher than in the previous two periods, whereas the weekly volume of patients on NET returned to normal.

**Table 1:** Characteristics of cohort by time period of diagnosis

Characteristics	Pre-pandemic (*N* = 21,027)	Immediate pandemic (*N* = 1570)	Peri-pandemic (*N* = 14,923)	*p*-value
Age at surgery	63.0 (52.0–72.0)	60.5 (50.0–71.0)	63.0 (53.0–72.0)	< 0.01
Female	20,848 (99.1)	1560 (99.4)	14,809 (99.2)	0.49
*Stage*				
1	13,776 (65.5)	914 (58.2)	9173 (61.5)	< 0.01
2	3308 (15.7)	281 (17.9)	2629 (17.6)	
3	1561 (7.4)	150 (9.6)	1116 (7.5)	
4	1084 (5.2)	110 (7.0)	822 (5.5)	
Unknown	1298 (6.2)	115 (7.3)	1183 (7.9)	
*Receptor*				
ER/PR+, HER2-	14,785 (70.3)	1034 (65.9)	10,128 (67.9)	< 0.01
HER2+	2989 (14.2)	260 (16.6)	2163 (14.5)	
Triple negative	1984 (9.4)	177 (11.3)	1404 (9.4)	
One or more biomarkers missing or unknown	1269 (6.0)	99 (6.3)	1228 (8.2)	
*First treatment*				
Neoadjuvant chemotherapy	3034 (14.4)	381 (24.3)	2907 (19.5)	< 0.01
Neoadjuvant endocrine therapy	1388 (6.6)	195 (12.4)	1020 (6.8)	
Surgery	15,311 (72.8)	882 (56.2)	9992 (67.0)	
No record of treatment	1294 (6.2)	112 (7.1)	1004 (6.7)	

Data are presented as median (interquartile range) or n (%) unless otherwise indicated

ER, estrogen receptor; HER2 −/+, human epidermal growth factor receptor 2 negative/positive; PR, progesterone receptor

**Fig. 3:**
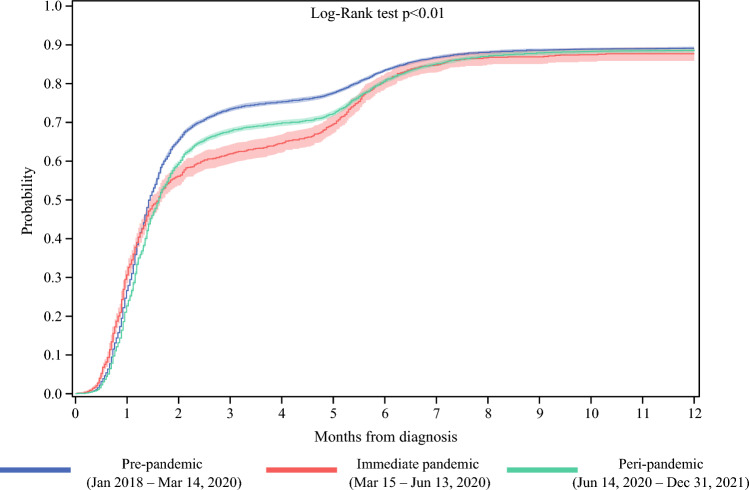
Cumulative incidence of having surgery from date of diagnosis for the entire cohort of patients with invasive breast cancer diagnosed between January 2018 and December 2021 (*N* = 37,520)

Figure 3 shows the cumulative incidence of having surgery within 12 months after diagnosis. The probability of all patients with BC undergoing surgery within 8 weeks from their date of diagnosis was 65.1% (95% confidence interval [CI] 64.4–65.7) in the pre-pandemic period, 56.1% (95% CI 53.6–58.6) in the immediate pandemic, and 59.1% (95% CI 58.3–59.9) in the peri-pandemic (log-rank *p* < 0.01).

However, these wait times varied according to the sub-cohort of treatment, including receipt of systemic therapy.

### Upfront Surgery

**Fig. 4:**
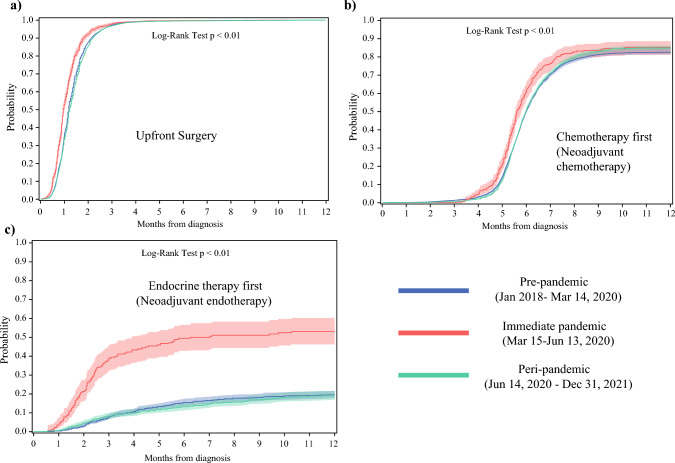
Cumulative incidence of surgery from date of diagnosis for patients with invasive breast cancer diagnosed between January 2018 and December 2021 stratified by first treatment

Figure 4a shows the cumulative incidence for patients with BC diagnosed between January 2018 and December 2021 who underwent surgery as their first treatment. The probability of patients undergoing surgery within 8 weeks of diagnosis was higher for patients diagnosed during the immediate pandemic at 92.1% (95% CI 90.2–93.7) compared with the 87.0% (95% CI 86.4–87.5) for patients diagnosed in the pre-pandemic period and 85.1% (95% CI 84.4–85.8) for peri-pandemic (log-rank *p* < 0.01).

### Neoadjuvant Chemotherapy

Figure 4b shows the cumulative incidence frequency of breast surgery for patients who underwent chemotherapy as their first treatment. Similar to the upfront surgery group, the probability of patients with BC undergoing surgery each month from their date of diagnosis was significantly higher in the immediate pandemic time frame but was similar to the peri-pandemic period compared with patients diagnosed in the pre-pandemic period (log-rank *p* < 0.01).

At 6 months from date of diagnosis, the probability of surgery for patients who received NAC was 50.2% (95% CI 48.4–52.1), 60.7% (95% CI 55.8–65.7), and 50.8% (95% CI 49.0–52.6) for patients diagnosed in the pre-, immediate, and peri-pandemic periods, respectively.

### Neoadjuvant Endocrine Therapy

Figure 4c shows the cumulative incidence of surgery among patients who underwent NET. The probability of this group of patients undergoing surgery each month from their date of diagnosis was significantly higher for patients in the immediate pandemic period than in the pre-pandemic period (log-rank, *p* < 0.01). However, the probability of surgery among patients diagnosed in the peri-pandemic period was similar to that for those diagnosed in the pre-pandemic period.

In total, 31,844 patients with BC (84.9%) underwent surgery during the study period. Wait times for surgery for all three groups (NAC, NET, and surgery first) were significantly lower during the immediate pandemic (in months, NAC 5.5 [IQR 5.0–6.1], NET 2.2 [IQR 1.6–3.0], surgery first 1.0 [IQR 0.7–1.3], *p* < 0.01) than during the pre-pandemic period (NAC 5.8 [5.2–6.4], NET 3.5 [2.2–5.6], surgery first 1.2 [0.9–1.6], *p* < 0.01) (Table 2). However, the absolute difference was relatively small, in the order of approximately 9 days for the NAC group and 6 days for the upfront surgery cohort. Wait times returned to baseline in the peri-pandemic period (NAC 5.8 [5.3–6.5], NET 3.5 [2.0–6.7], surgery first 1.2 [0.9–1.7], *p* < 0.01).

**Table 2:** Median wait time for surgery from date of diagnosis (in months) and interquartile range by time period for subcohort of patients who had surgery (*N* = 31,844)

Characteristics	Pre-pandemic (*N* = 17,991)	Immediate pandemic (*N* = 1,294)	Peri-pandemic (*N* = 12,559)	*p*-value
Neoadjuvant chemotherapy (*n* = 5119)	5.8 (5.2–6.4)	5.5 (5.0–6.1)	5.8 (5.3–6.5)	< 0.01
Neoadjuvant endocrine therapy (*n* = 185)	3.5 (2.2–5.6)	2.2 (1.6–3.0)	3.5 (2.0–6.7)	< 0.01
Surgery first (*n* = 26,185)	1.2 (0.9–1.6)	1.0 (0.7–1.3)	1.2 (0.9–1.7)	< 0.01

## Discussion

In this large population-based study, we described wait times for BC surgery across Ontario, Canada, during the COVID-19 pandemic time frame compared with baseline. Although patients diagnosed with BC during the immediate pandemic experienced longer wait times between diagnosis and surgery (compared with pre- and peri-pandemic periods), this difference coincided with a shift toward prioritizing surgery in patients who had already completed neoadjuvant systemic therapy (endocrine or chemotherapy). When stratified by first treatment, the median wait time during the immediate pandemic was slightly shorter than for patients diagnosed in the pre-pandemic period. Importantly, these data also show that wait times to surgery for patients who had NAC during the immediate pandemic were preserved.

The median wait time to surgery for patients with BC was slightly shorter during the immediate pandemic period than during the pre-pandemic period. We hypothesize that this is likely multifactorial: given the mainly outpatient nature of breast surgery, many breast procedures were prioritized for operative time despite pandemic-related restrictions for other types of surgeries. Furthermore, fewer patients with BC may have been diagnosed during this period, leading to a lower volume of patients requiring surgical treatment. Finally, patients undergoing NAC would have been prioritized for surgery, ensuring—as much as possible—surgery within the recommended post-chemotherapy time frame. Our findings are similar to those from other studies. For example, a study from British Columbia, Canada, showed that, during the immediate pandemic, time from core biopsy to surgery reduced from 58 days to 28 days^13^ and a study of data for 895,116 female patients diagnosed between 2018 and 2021 from the National Cancer Database showed an average decrease in wait times of about 5.5 days between January 2020 and May 2020.^14^ This shorter wait time during the immediate pandemic period seems to be unique to BC surgeries, as delays for other oncologic surgeries during COVID-19 are well described: A population-based study in Canada showed that the mean wait times for all cancer surgeries increased from 25 days pre-pandemic to 32 days during the first 6 months of the pandemic,^15^ suggesting that not all surgical oncology care was affected uniformly by the pandemic-induced changes in the health care system. BC surgeries were likely allowed to continue while other cancer surgeries were postponed during the immediate pandemic as they required more minimal resources in acute care hospitals. Specifically, most BC surgeries, even larger operations such as mastectomy with reconstruction, can be safely performed in an outpatient setting^16,17^ and with better patient satisfaction.^18^ This transition to more outpatient BC surgery was facilitated by the increasing use of regional anesthesia^19^ and implementation of various protocols to streamline postoperative recovery during the pandemic if appropriate.^20,21^

While the absolute difference in surgical wait times between patients in our three time cohorts is small (approximately 1 week) and unlikely to affect oncologic outcomes, this difference in time may be significant from a patient perspective. It has been well documented that patients with BC experience immense anxiety while waiting for surgery.^22^ This anxiety was likely exacerbated by greater uncertainty during the first few months of the COVID-19 pandemic.^23^ Therefore, a shorter wait time for surgery may have been even more important for patients. Although the surgical wait times for patients with BC did increase and return to pre-pandemic levels during the peri-pandemic period, this was still achieved despite higher volumes of COVID-19 inpatient admissions resulting in ongoing surgical resource limitations.^24^

A higher proportion of patients were placed on NAC and, importantly, trials of NET during the immediate pandemic period. This increase in the use of neoadjuvant systemic therapy may have been in response to limited operating room and health care resource availability necessitating surgeons and medical oncologists to triage operative resources and develop treatment plans based on tumor biology. For example, patients with early-stage hormone receptor positive, HER2- BC typically undergo upfront surgery; however, during the immediate pandemic, the use of genomic testing on core biopsies^25^ (rather than the final surgical specimen) increased, suggesting that physicians wanted a better understanding of tumor biology to manage treatment pathways. Our findings are similar to those from a Canadian study of neoadjuvant systemic therapy use during the pandemic, which showed that patients were more likely to start on upfront systemic therapy (either endocrine or chemotherapy) in the first few months of the pandemic but that there were regional variations in the likelihood of receiving systemic therapy.^26^ Here, we have not only identified the neoadjuvant practice patterns during the immediate pandemic period but also encompassed a longer follow-up time by including the peri-pandemic phase, encompassing multiple “waves” contributing to ongoing COVID-19 hospitalizations across Ontario. These data showing a return to pre-pandemic levels of NET usage in Ontario after the immediate pandemic phase are similar to findings of a large National Cancer Database study on BC management during the pandemic.^14^

The proportion of patients on NAC was higher during the immediate pandemic and peri-pandemic period than in the pre-pandemic period. This may be because a higher proportion of patients were diagnosed with triple negative or HER2+ BC tumours during the immediate pandemic than during the pre- and peri-pandemic periods; these subtypes more often present as palpable abnormalities as compared with screen-detected findings, reflecting their more aggressive nature. This relative shift in biomarker profile may reflect that fewer hormone receptor-positive/HER2- BCs were diagnosed during this period of suspension of screening. Previous studies have shown that the majority of screen-detected tumours are hormone sensitive and HER2-, whereas non-screen-detected tumors are more likely to be triple negative or HER2 enriched.^27^ In Ontario, BC screening was stopped for 2 months between March 23, 2020, and May 31, 2020,^1^ with gradual resumption of screening beginning with high-risk patients. The program resumed full capacity in January 2021 when eligible patients were offered the opportunity to participate in screening again. However, the increased use of NAC may also be indicative of higher-stage disease presentations; further research is needed to understand the impact of health care changes on BC screening and disease presentation. We also identified that a higher proportion of patients were treated with NET during the immediate pandemic period than at baseline; this treatment approach returned to pre-pandemic levels in the peri-pandemic period. This was likely because triage guidelines published during the COVID-19 pandemic^4^ recommended that patients with hormone-sensitive, HER2- tumors who are not otherwise candidates for NAC be placed on NET to preserve operating room capacity and limit exposure to COVID-19. However, despite the burden on the health care system with periods of increased COVID-19 hospitalization during the peri-pandemic period, the number of patients redirected to endocrine therapy as the first treatment during the immediate pandemic did not increase. This suggests that lessons were learned in preserving access to surgical care for patients who had early-stage hormone-sensitive BC. The return to baseline rates of NET in Ontario likely reflects the fact that NET is usually reserved for selected patients with ER+, HER2- BC who are unresectable, unfit for surgery, or interested in downstaging their disease to allow for breast-conserving surgery but are not good candidates for chemotherapy.^28^

### Limitations

This study is limited by the retrospective study design and use of administrative health care datasets. Specifically, it does not capture detailed information about tumor size or nodal size, making it challenging to extrapolate whether the reason for the increased NAC use was due to a higher stage at time of diagnosis. We identified no clinically significant delay to surgical therapy but did see a change in how BC was managed during the immediate pandemic with respect to increased NET and NAC utilization. Although further information regarding long-term oncologic outcomes are not yet available, we have identified a cohort of patients with BC who can be followed to investigate further. Lastly, the data presented here are not granular enough to reflect variations in surgical wait times across specific Ontario centers. A previous study of time to surgery in a multicenter, publicly funded hospital system identified a slight increase in time to surgery during the pandemic, driven mainly by one hospital that had a wait time increase from a median of 42–129 days, whereas the remaining two hospitals had decreases in their time to surgery.^29^ We plan to review regional variations in BC surgery across all provincial health regions in a follow-up study to understand inequities and to develop strategies to reduce their impact during times of public health emergency.

## Conclusion

Although the time between diagnosis and surgery was slightly longer for all patients during the first few months of the COVID-19 pandemic, a higher proportion of patients were treated with NET or NAC. When stratified by treatment, wait times were relatively similar for patients undergoing surgery first and, importantly, surgical wait times for patients undergoing NAC were preserved. The long-term impact of changes in the management of BC on oncologic outcomes of patients diagnosed during the immediate COVID-19 pandemic remains unknown. Further studies exploring these outcomes will benefit our understanding of health care systems’ responses to global health crises.

## Supplementary Information

Below is the link to the electronic supplementary material.

Supplementary file1 (DOCX 24 KB)

## Data Availability

The dataset from this study is held securely in coded form at ICES. Although legal data-sharing agreements between ICES and data providers prohibit ICES from making the dataset publicly available, access may be granted to those who meet pre-specified criteria for confidential access, available at www.ices.on.ca/DAS (email: das@ices.on.ca). The full dataset creation plan and underlying analytic code are available from the authors upon request, understanding that the computer programs may rely upon coding templates or macros that are unique to ICES and are therefore either inaccessible or may require modification.
