# Supplementary material for: Breast Cancer Surgery Wait Times and COVID-19: A Canadian Population-Based Analysis
Source: Ann Surg Oncol. 2026 Jan 25;33(5):4472–81. doi: 10.1245/s10434-025-19029-3 (PMC13083350; doi:10.1245/s10434-025-19029-3)
Supplement: Supplementary file 1 — Supplementary file1 (DOCX 24 KB) [file 10434_2025_19029_MOESM1_ESM.docx]

Supplementary Table 1. Data Sources from ICES

| **Database** | **Description** |
| --- | --- |
| Ontario Cancer Registry (OCR) | The OCR is a passive, provincial registry all incident cancer diagnoses in Ontario. It includes 96% of cancer diagnoses in the province. Information included in the registry: cancer topography and morphology/histology, and details on diagnosis (e.g., types of contributing information to the diagnosis, dates). |
| Registered Patient Database (RPDB) | The RPDB is an ICES database derived from all administrative data sources and provides demographic data including age, patient residence, vital status, date of last contact with the healthcare system, and OHIP eligibility. |
| Ontario Registrar General (ORG) | The ORG contains gold standard vital status data for all Ontarians. According to the Vital Statistics Act, it is mandatory to register all deaths occurring in the province. |
| Canadian Institute of Health Information – Discharge Abstract Database and Same-Day Surgery (CIHI-DAD and SDS) | CIHI-DAD and SDS are mandatory reporting systems that provide information on hospital admissions and same-day surgeries, including diagnoses, procedures, and length of stay. |
| National Ambulatory Care  Reporting System (NACRS) | The NACRS collects data elements describing emergency health services provided by EDs in Ontario, including diagnoses, procedures, and administrative information such as wait times. |
| Activity Level Report (ALR) of Cancer Care Ontario | Cancer Care Ontario maintains a database of cancers-specific services, including consultations, chemotherapy, and radiotherapy provided by regional cancer centers in the province. Because all radiotherapy is delivered at Regional Cancer Centres (RCC), it is a complete source of information. However, because not all cancer patients who receive surgery or chemotherapy visit an RCC, it cannot be used as a population data sources for those treatments. |
| Ontario Marginalization Index (ONMarg) | The ON-MARG is a specialized database using Census data to profile relative area-level marginalization dependency, deprivation, ethnic concentration, and instability at various geographic levels in Ontario. |
